# Mechanical Testing of Composite Steel and Reactive Powder Concrete Structural Element

**DOI:** 10.3390/ma13183954

**Published:** 2020-09-07

**Authors:** Jan Bujnak, Peter Michalek, Frantisek Bahleda, Stefania Grzeszczyk, Aneta Matuszek-Chmurowska, Arkadiusz Mordak

**Affiliations:** 1 Faculty of Civil Engineering, University of Žilina, Univerzitná 78215/1, 010 26 Žilina, Slovakia; peter.michalek@uniza.sk (P.M.); frantisek.bahleda@uniza.sk (F.B.); 2 Faculty of Civil Engineering, Opole University of Technology, Katowicka 48, 45 061 Opole, Poland; s.grzeszczyk@po.edu.pl (S.G.); a.matuszek-chmurowska@po.edu.pl (A.M.-C.); a.mordak@po.edu.pl (A.M.)

**Keywords:** reactive powder concrete, material property test, composite element, numerical modeling

## Abstract

Reactive powder concrete (RPC), typically with higher compressive strength, is particularly attractive to structural engineers to apply them in infrastructures for enhancing their resistance under severe environments and loads. The main objective of the initial study presented in the paper was to investigate the behavior of two types of these new cementitious materials differing in the nature of microfibers. The RPC mixes were reinforced with steel and then with basalt microfibers. To evaluate the structural performance of developed unconventional materials, properties were investigated experimentally and compared with the control normal concrete mix. Mechanical tests indicated that dispersing fine fibers for making RPC, a mean compressive strength of 198.3 MPa and flexural strength 52.6 MPa or 23.2 MPa, respectively, were developed after 28 days of standard curing at ambient temperatures. In composite structures consisting of steel girders and a concrete slab, it is necessary to prevent the relative slip at the steel and concrete interface using shear connectors. The very high RPC strength enabled a material saving, weight-reduced application of precast construction, and particularly effective joint to steel beams. The investigation of such shear connection efficiency, in the case of the higher strength concrete deck, using standard push-out test specimens was executed. Finite element numerical models were developed. The outputs of the studies are presented in the paper.

## 1. Introduction

Reactive powder concrete (RPC) is one of the most advanced cement-based composite materials in the group of ultra-high performance concretes (UHPC). Such composites can provide a compressive strength above 140 MPa, usually about 200 MPa, as already observed by Richard [1], Aïtcin [2], and Zdeb and Śliwiński [3]. In these innovative concrete types, very high material strength is achieved by replacing coarse aggregate with finely ground quartz of grain sizes from 1 µm to 4 µm and sand with grains from 0.2 to 0.4 mm. Improved homogeneity and compactness can be achieved through the ideal grading by adding also silica fume. By using superplasticizers, it is possible to reduce water and cement ratio (w/c). Its low value of about 0.2 ensures that a significant part of the cement is not hydrated. Improved granular compactness through the use of powders with a complementary grain size distribution, including silica fume, eliminates the presence of a transition zone between sand and slurry particles and ensures very low porosity of the material.

A new group of RPC composites is reinforced with short, thin steel fibers, usually 0.2 mm in diameter and 12 mm long emerged, in a quantity of approximately 2% by volume. As a result of further research in the 1990s, one of the first composite, according to [4], was called Ductal, which means a whole family of composites with excellent properties, with a compressive strength going from 180 MPa to 230 MPa, and flexural one is ranging between 30 MPa and 50 MPa. The composite is also characterized by high tensile strength, excellent impact, as well as abrasion resistance and can provide extraordinary durability [5]. Potential Ductal modeling and testing were specified by Adeeb et al. in [6]. Examples of construction applications of this bendable composite can be found also in [7]. But soon, many other commercial products of this type were documented by Zych [8]. In this study, especially unconventional and more environmentally resistant basalt fiber’s effects on RPC properties were examined and compared to steel admixtures.

The favorable properties of RPC composites permit to create thin profiles and to overspan considerable structural areas. Aïtcin [9], Perry and all [10], Li and Kanda [11] illustrated in an exemplary way light and slender structures as well as innovative structural forms, simultaneously durable and resistant to corrosion. Moreover, precast lighter elements, compared to massive and heavy structural parts made of traditional materials, are preferred today. Blais and Couture [12] illustrated the application of precast RPC elements of complex shapes, which are only 20 mm thick for the facade, roof, and coating of buildings. Mazzacane et al. [13] gave details on the roof of Jean Bouin stadium in Paris, consisting of 3600 precast RPC panels, only 35 mm thick, covering an area of 23,000 m^2^.

Buildings constructions are often in need of spanning greater distances between vertical supports. Composite steel beams cooperating with the concrete flooring deck, placed on the upper flange, represent a preferable solution. The interiors of the building can be better used without restrictive vertical columns. In particular, trusses may allow the placement of various actually required several installations into the free space between the cords. Besides, steel-concrete composite bridges are used as an alternative to ordinary structural systems because of their ability to adapt their geometry to design constraints and the possibility of using favorable properties of both structural materials [14]. However, in the case of presently preferable truss main girders, the transmission of longitudinal forces takes place only discontinuously in the nodes, where the web members are connected to the compressed chord [15]. But in actually available specifications, this design situation is treated only rather approximately. The phenomenon of slippage at the steel-concrete interface depends on many factors, including the type and size of the shear connectors, their spacing, the type of concrete slab, and the strength of the concrete at the location of the connectors. Combining shear connectors and RPC in these locations can be an effective solution to prevent the relative slip at the steel-concrete interface when particularly precast deck is used. As it may be uneconomical to make a concrete slab entirely of such concrete, the use of RPC to fill element joints with steel shear connectors seems greatly appropriate. By increasing joint stiffness, higher composite effectiveness in steel-concrete elements can be achieved, which has also been confirmed by other authors [16,17].

Thanks to its properties, RPC is increasingly being used in widely understood composite structures in which layers of concrete of different classes are joined together. This allows for the strengthening of existing structures [18] but also for the production of composite structural elements with less weight and dimensions and interesting mechanical properties [19]. 

## 2. Preliminary RPC Properties Study

### 2.1. Mixes and Sample Structure

For this introductory study, three groups of mixes were prepared. The control concrete series (C) examined the samples’ behavior without fibers. Two new RPC sets contained steel fibers (S) or unconventional basalt fibers (B). Portland cement CEM I 52.5 R was produced in Polish cement plant Rejowiec. Moreover, ingredients and admixtures were added for achieving a specific surface area 410 m^2^/kg as well as a very high initial strength. Silica quartz powder 0–0.2 µm from Łaziska ironworks and silica sand 0–0.4 mm from Osiecznica stone quarry were also used as additives. This study involved two different RPC materials. At first, the steel fibers 12 mm long and 0.2 mm in diameter were spread in the mixture for the development of RPC. Basalt fibers of 12 mm length with 18 µm thickness were used for the other innovative testing. A polycarboxylate superplasticizer was added at a ratio of 2.5% of cement weight to fluidify the mixture. The chemical composition of cement, silica fume, quartz powder, and quartz sand is given in Table 1. This analysis was conducted by means of spectrometer analytical Axios Minerals+ (Malvern, UK) via the X-ray fluorescence spectroscopy method with wave dispersion.

**Figure 1:**
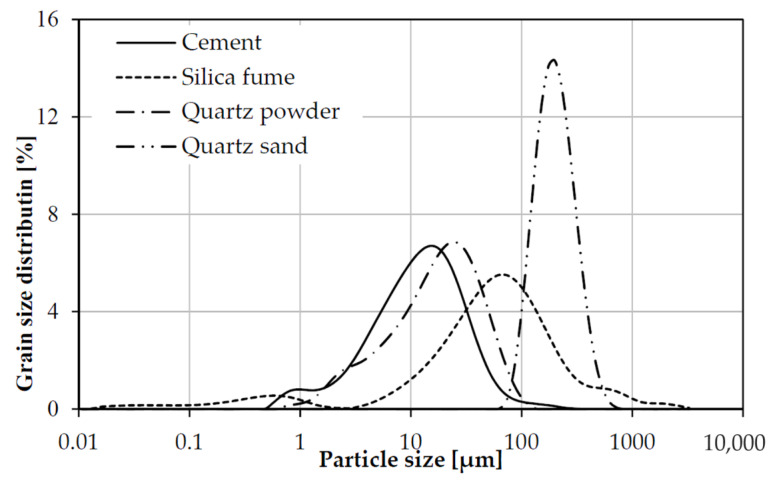
Grain composition of cement, silica fume, quartz powder, and quartz sand.

The grading of fine materials in dry conditions used for the entire mixtures is shown in Figure 1. The particle size distribution of the concrete was determined by means of the laser analyzer Mastersizer 3000 (Taren Point, Australia) within the range 0.01–3500 μm. The optimization of the concrete mixture composition to increase the degree of grain packing was based on the Funk and Dinger curve [20].

The control concrete samples without fibers (C) and RPC sets containing steel fibers (S) or unconventional basalt fibers (B) composition is shown in Table 2.

### 2.2. Assessment of Concrete Mix 

The studies of concrete and RPC mixtures consistency were performed according to CEN-EN 1015-3 standard [21], based on the measurement of the mixture flow diameter. The test results after 15 min, using the flow table method, are given in Table 3. The results of consistency tests of concrete mixes made of RPC showed that the diameter of the flow was between 230 and 250 mm, which allowed for the easy casting of the RPC mix. As can be seen from the data in Table 3, the introduction of basalt fibers into the mix caused the greatest loss of flowability. 

The concrete density tests were carried out in accordance with the standard EN 1015-10 [22]. The density was determined on the basis of the mass of the dried sample and its volume in the saturated state when immersed in water. The results of the density tests of concrete made of reactive powders showed that the density in the dry state was between 2280 and 2590 kg/m^3^ as given in Table 4. The S concrete had the highest density, which was due to the considerable amount of steel fibers in this concrete.

The flexural and compressive strength were determined according to the EN 1015-11 standard [23]. The 40 mm × 40 mm × 160 mm bars were produced. After 24 h storage of the molds at standard conditions, i.e., temperature 20 ± 2 °C, relative humidity 60 ± 5%, the bars were demolded and immersed in water at temperature 20 ± 2 °C. The compressive strength tests were executed after 2, 7, and 28 days of storage in water. The results of compressive and flexural strength tests of concrete are summarized in Table 5. From the data contained in Table 5, it is evident that the compressive strength of RPC with steel fibers achieved 145.4 MPa after only two days of curing, while after 28 days, it reached about 198.3 MPa. The compressive strength of RPC with basalt fibers was significantly lower at the amount of 88.7 MPa or 135.6 MPa, respectively.

The freeze-thaw resistance of concrete in the presence of deicers (3% NaCl) was determined according to the rules given in the CEN/TS 12390-9 document [24]. The results of frost resistance tests of RPC after 56 freezing and defrosting cycles in the presence of deicing salts are presented in Table 6. RPC concretes (C, S, B) showed very high resistance to frost and deicers. Scaling of concrete samples after 56 cycles was only 0.0002 kg/m^2^ to 0.0007 kg/m^2^. In the case of RPC, this value was negligibly higher. When basalt fibers were used, the mass loss appeared even to be smaller. This allowed the frost resistance of RPC concrete to be assessed as very good material according to the criteria adopted by the standard, regardless of the type of fibers used. 

The porosity of concrete samples was measured using the mercury PoreMaster 60 porosimeter under pressure in the range from 1 to 400 MPa [25]. The analysis of RPC concrete porosity test results is presented in Table 7. 

As can be seen from the data in Table 7, the total pore volume content of RPC concrete samples after 28 days of curing could increase with the addition of fibers in the concrete. The addition of basalt fibers caused a slight increase in the total porosity of RPC from 4.4% (RPC without fibers) to 5.4%. On the contrary, the addition of a significant amount of steel fibers could result in a significant increase in the total porosity at 12.4%. In RPC concretes, without fibers and with basalt fibers, the pore size distribution was very similar. There was a clear prevalence of mesoporous below 20 nm in diameter (around 77%), while the share of pores above 20,000 nm in diameter for RPC concrete with basalt fibers (B) was increasing. This was not the case for RPC concretes with a large number of steel fibers, where the total pore volume content was significantly higher than for RPC without fibers and with basalt fibers. In this case, the number of mesoporous, smaller than 20 nm in diameter (15.7%), was much smaller, while the proportion of pores between 20 and 200 nm in diameter was larger (77.0%). The above properties of RPC were achieved only by the appropriate selection of components.

## 3. Testing of Composite Element

### 3.1. Concrete Parts Properties of Composite Samples

For assuring the connection of steel beams and a concrete slab, steel stud connectors are normally applied. Potential employment of the RPC to attach particularly precast concrete decks to steel superstructure was the subject of the subsequent study. The shear capacity and the load-slip relations are the most important characteristics for the design of this type of composite structure. The suitable method to find the shear connection properties could be full-scale composite beam tests, but they are quite time-consuming and rather costly [26]. Therefore, a push-out test was implemented in the research [27]. During this experimental investigation, the local shear force was applied to a concrete specimen. The transfer of the resulting shear force between the steel section and concrete slabs was provided by shear studs encased in RPC.

**Figure 2:**
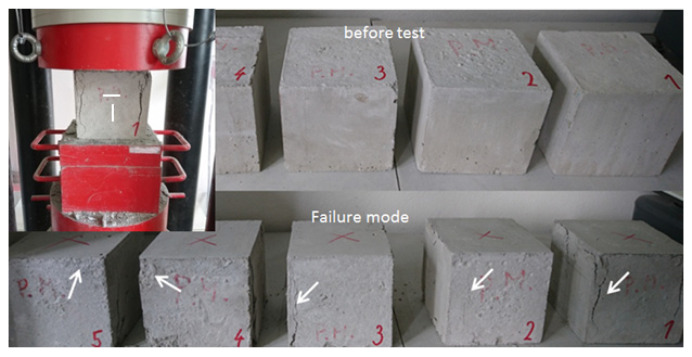
The concrete specimen in the testing machine with cracks appeared.

The experimental work started in mixing, casting, and testing the mechanical properties of ordinary and high-strength concrete. Consequently, thus, three sets of specimens for mechanical properties testing were prepared to determine the compressive and flexural strength as well as elastic modulus of ordinary concrete but also RPC with the addition of steel or basalt fibers. The mechanical performance of the produced RPC was primarily considered by compressive strength, evaluated by exchanging different fibers according to EN 12390-3 [28]. Three sets of concrete cubes (150 mm × 150 mm × 150 mm) were prepared. Each set consisted of three specimens, cast in steel molds, and demolded 24 h later. Figure 2 gives the concrete specimen on the testing machine producing predominantly compression.

The mean compressive strengths of six specimens produced from both categories of RPC, as well as the values of three ordinary concrete samples, are summarized in Table 8. The experimental values showed only slight variations of the fibers-reinforced materials’ strength. The average round strength value could be considered as 120 N/mm^2^. The studies even confirmed that incorporating microfibers into the mixtures resulted in higher early compressive strength. 

**Figure 3:**
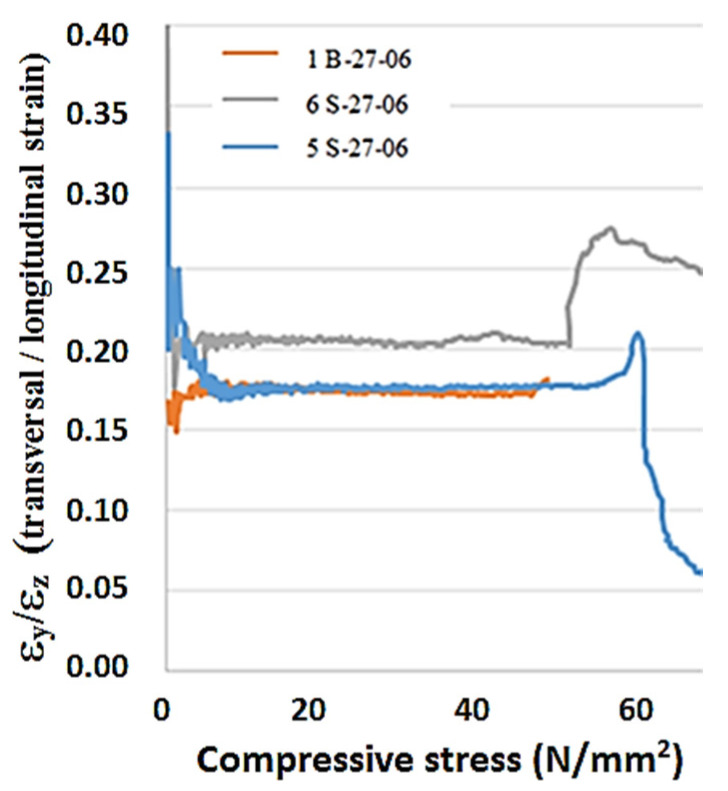
Poisson’s ratio as a function of compression.

The cube specimens were also used to determine other material characteristics. Although a different aspect ratio of the samples compared to the cubes would be more appropriate to exclude the influence of the dimensions. They were instrumented with four strain gauges on the opposite sides. Two of them were used for vertical strain measurement and the further two for transversal horizontal strain recording. During the compressive test, the load history was measured by a universal testing machine, while the strains of the specimen were obtained by averaging two sideways gages values, as shown in Figure 2. Thus, Poisson’s ratio could be identified. An example of its values as load function is given in Figure 3. In the linear range, this ratio rose from 0.18 to 0.20. 

**Figure 4:**
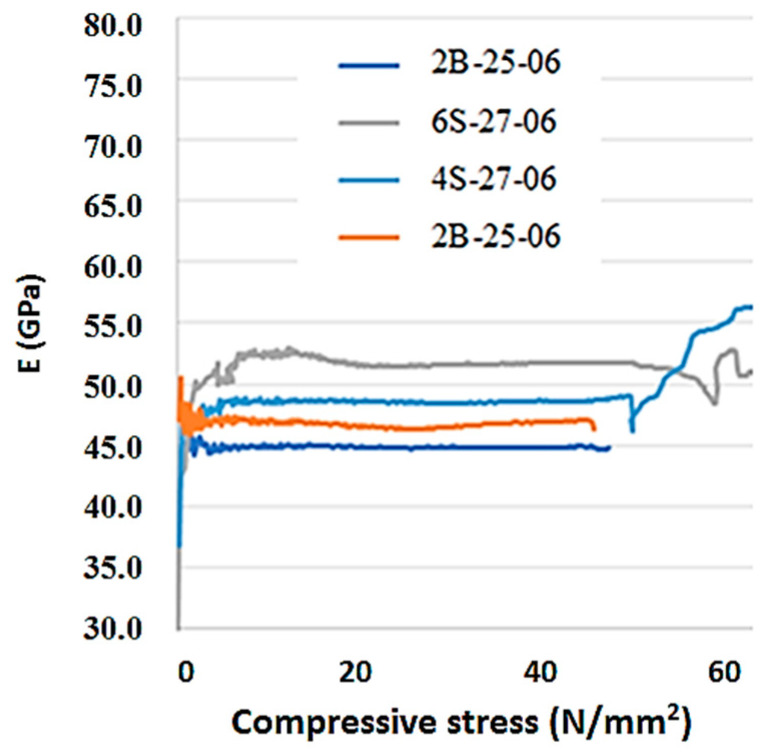
Load-modulus experimental curves.

The Young’s modulus *E* computed with the longitudinal strains on the elastic part of the curve in Figure 4 was 54 GPa, and in the case of basalt microfibers, slightly less, 47 GPa. Damages, accompanied by a reduction in the modulus values, only have occurred above 50% of the ultimate average compressive strength.

**Figure 5:**
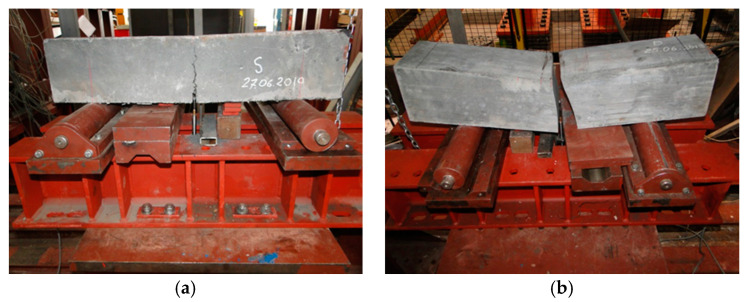
Specimens during the flexural strength test (**a**) and when failed (**b**).

The tensile behavior could be determined from the load-deflection curve obtained by applying a center-point load on a simply supported notch prism. It tested the ability of unreinforced concrete beam to withstand failure in bending. The test specimens were two prisms per composition, totaling six ones conforming to EN 12390-1 [29], with standard width and depth of 150 mm and the length 600 mm. In Figure 5, test arrangements and testing for the flexural strength of the beam in the laboratory can be observed. The device for transmitting the load of the testing machine to the test specimen consisted of two supporting rollers and one loading pin. The span length as the distance between the centers of the supporting rollers was equal to 500 mm. The testing machine had applied loads at a uniform rate and continuously without shock until the point of failure at a constant rate of 4 kN/min. The wet sawing was used to notch the test specimens in the mid-span section. The width of the notch was 4 mm. Linear displacement transducers with a measured displacement accuracy of 0.01 mm were installed in the mid-span to register specimen deflections. 

The experimental force-deflections curve for specimens reinforced with steel microfibers consisted, generally from the linear-elastic part of the diagram, up to 75% of the applied load. The progressive tensile damage beyond this point made the curve diverge from the straight line. Nevertheless, microfibers reinforcement could redistribute the stress between initiated microcracking, which did not lead to precipitate failure of the specimen. But multiple propagated microcracks ended at the peak of the load-deflection curve. The strains were localized in a single crack opening in the mid-span, where the final failure occurred. The average flexural strength on all RPC standard beams specimens was 25.46 N/mm^2^. The application of fibers in conventional concrete as supplementary cementitious material to an appropriate amount was seen and exhibited better mechanical behavior.

### 3.2. Testing of Push-Out Specimens

**Figure 6:**
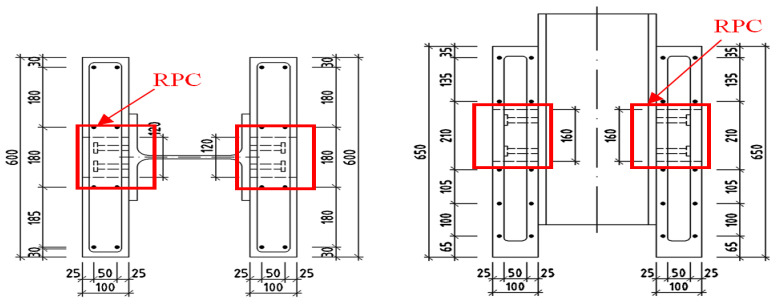
Push-out specimen of RPC with steel *S1–2* and basalt *B1–2* fibers.

The push-out investigation was executed in accordance with EN 1994 [30,31]. As in the similar experimental testing [32], the specimens consisted of a steel shape *HEB 260B* and two concrete slabs attached to the flanges of the steel beam. The concrete slabs were 650 mm long, 600 mm wide, attached using four stud connectors with a shank diameter of 16 mm and 75 mm in height to each flange. The longitudinal distance between the connectors was 96 mm, and the transversal pitch 56 mm. Two test specimens, *C1* and *C2,* included both slabs of the ordinary concrete *C25/30.* In the case of specimens *S1* and *S2*, the RPC mortar was added, a blend of micro smooth steel fiber. The specimens *B1* and *B2* were prepared with the addition of basalt fibers. As shown in Figure 6, shear connections were completed by casting and filling in the square holes with RPC mortar of compressive strength of 120 MPa.

**Figure 7:**
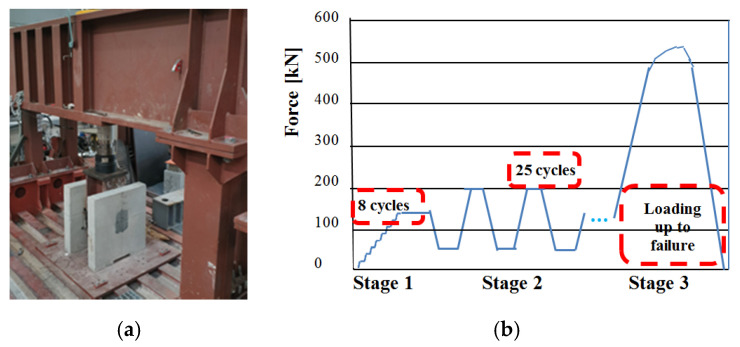
Layout of push-out specimen of RPC (**a**); loading procedure (**b**).

**Figure 8:**
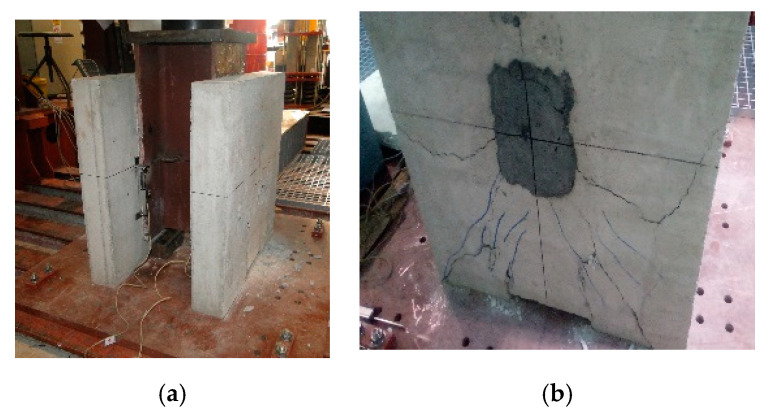
The specimen in the testing machine (**a**) and ultimate failure cracking (**b**).

Push-out specimens were kept over a steel base plate. The contact surfaces of steel and concrete were cleaned and smoothened to avoid eccentric loading. The specimens were tested using a universal testing machine until complete failure. Dial and strain gauges were mounted at critical locations, as shown in Figure 7 and Figure 8. The test procedure began with load increments of 20 kN up to 160 kN, a value representing about 40% of expected ultimate carrying capacity for checking the performance of the instruments mounted at the specified locations. This was followed by 25 repeated loads from 40 kN to 200 kN. Then, imposed displacement loading continued up to the failure at the speed of 1 mm in 2 min.

At each load or displacement increments, the slip history between two concrete slabs and the steel beam was measured by linear variable transformers and recorded. Its average value was plotted against the load per connector.

**Figure 9:**
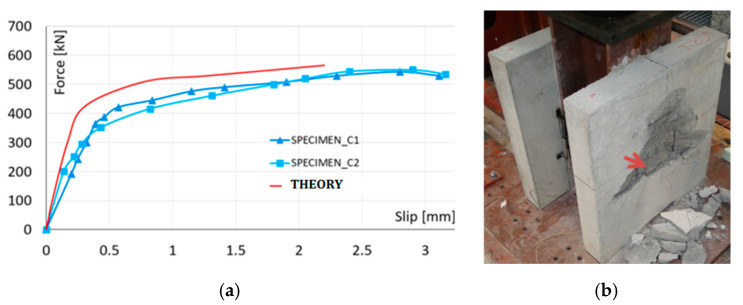
Load-slip curves of the ordinary concrete specimens (**a**) and failure mode (**b**).

The cracking patterns during each load increment were carefully observed to identify the failure mechanism. Interface slip between the steel flange and concrete slab was measured using dial gauges mounted by keeping the tip in a vertical direction. It was noted that during the initial 25 cyclings between 40 kN ens 200 kN, both specimens *C1* and *C2* remained in good condition without any cracking. In both push-out specimens, first of all, the interface cracks on the sides were observed at about 300 kN, thus around 55% of the ultimate failure loads. Cracks along the width of the concrete slab started appearing at the level of shear connectors beyond the load of 400 kN. Gradually, the cracking of the concrete slab caused the failure of stud shear connectors by fracture at an ultimate load of 543 kN, as illustrated in Figure 9, and the ultimate slip value was 3 mm. 

**Figure 10:**
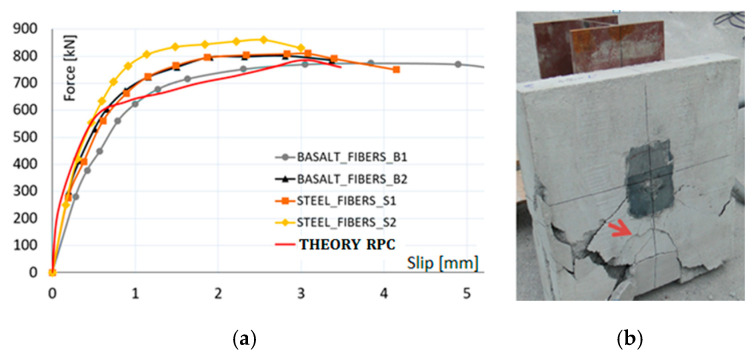
Load-slip curves of the RPC specimens (**a**) and failure mode (**b**).

In the second set of four RPC specimens, the failure of concrete slabs started due to the spalling of ordinary concrete at the corner of slabs, as shown in Figure 10. Gradually, the cracking of the slab caused the failure of stud shear connectors by fracture at the ultimate load. Experimental results testified that the average ultimate load, about 41% superior, was achieved in this study. It can be concluded from this experimental study that RPC in the vicinity of the stud significantly enhanced the compressive strength as well as splitting resistance of concrete.

### 3.3. Numerical Modeling of Testing

**Figure 11:**
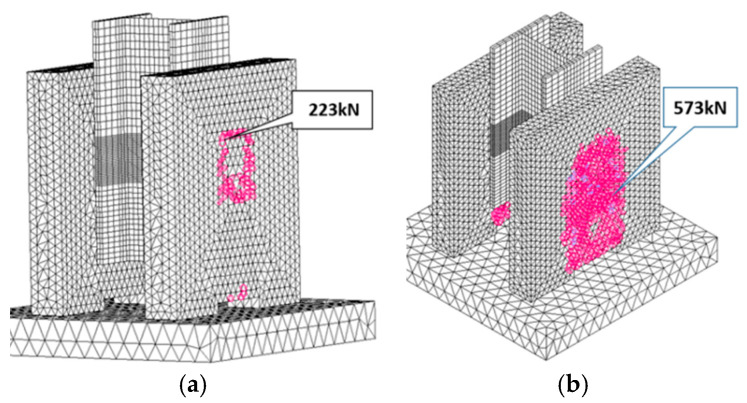
The ordinary concrete specimen theoretical cracking under relatively small intermediary loading (**a**) and at the final stage (**b**).

The push-out tests remained also the time-consuming and costly option. Therefore, numerical simulation was developed as a tool for the investigation of the behavior of the connection, using the least possible resources, to achieve an adequate relationship with reality. In this study, the numerical simulation process employed ADINA software, based on a finite element method. The tri-dimensional modeling of the specimen was adopted, thanks to the favorable geometrical representation provided by the software, which was consistent with the experimental test layout. Load increments were applied at small intervals, where the size of them was automatically selected by the software code, based on numeric convergence conditions. In order to model the steel strain-stress behavior, a bilinear behavior was adopted. The concrete damage plasticity model was employed, which was implemented in the ADINA code. This software offers a large program library with solid space elements with different typologies. The finite elements to be used for the discretization of concrete slabs and steel parts are illustrated in Figure 11. The adequate size to be used for discretization and the corresponding mesh density were determined by a special study comparing simulation with experimental tests. The circular studs were approximated by hexagonal strut elements of 0.005 m in diameter.

**Figure 12:**
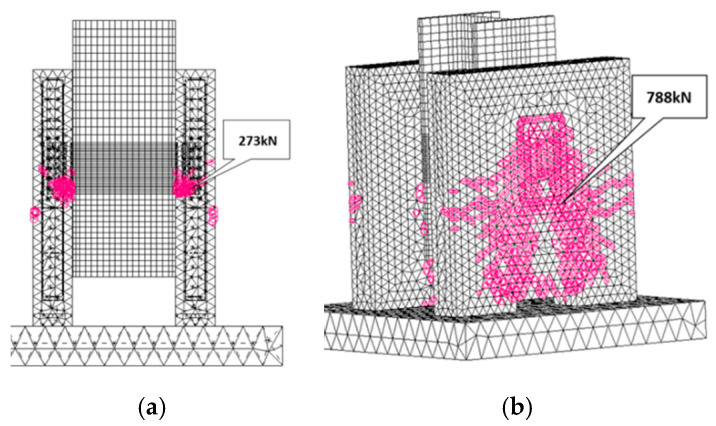
Theoretically different cracking of RPC specimens in intermediate stage (**a**) and at remarkably higher ultimate load (**b**).

Figure 10 shows the comparison between the load-slip curves obtained experimentally and numerically using the finite element method. The curve obtained from numerical modeling is called THEORY. Theoretical and experimental values of slip confirmed a good agreement between the test and the numerical model in the range of practically applied loading. The first crack in the concrete slab was initiated at the force value of 54 kN. The following cracking propagation, under small load up to 223 kN, did not modify linear behavior remarkably. The theoretically declared ultimate loading capacity was 573 kN. The corresponding damaged areas are plotted in Figure 12. 

For both types of RPC push-out specimens, the experimental slips, similarly, initially were increasing proportionally to the slightly higher loading level of 273 kN, as shown in Figure 12. However, with the increasing load, the diagrams presented new faster overall slip development. The slips under supplementary loading were around 3.5 mm at the end of the test and the ultimate force value of 788 kN, so by a third larger than in the case of ordinary concrete. Finally, the connection failed by cracking in ordinary concrete slab parts without RPC encasement local damages as well as no shear connector defects. Thus, the bending failure of connectors did not need to be taken into consideration due to favorable load transmission by RPC encasement. 

An adequate consistency between numerical and experimental results could be observed and proved by employing previously calibrated numerical simulation. Consequently, the use of the finite element model was validated. As a result of this study, it can be concluded that numerical modeling and experimental tests are complementary research tools.

## 4. Discussion of Results

The effects of processing additions were analyzed based on the work conducted. The composition of the RPC mix was analyzed using X-ray fluorescence spectroscopy. The data shown in Table 1 complied with the standard chemical requirements. The cement, silica fume, quartz powder, and sand were analyzed by laser. Examination of Figure 1 shows that there was some increase in quartz sand particles in the samples. Relatively significant effects of steel fibers appeared to be involved in the affected systems slightly more than the basalt ones. The mix parameters that seemed to be most sensitive to changing cementitious systems were compressive and flexural strengths, as shown in Table 5. The results observed in changing flowability in Table 3 indicated only a few important influences of admixtures. Similarly, there did not appear to be a substantial difference between mixed frost resistances summarized in Table 6. None of the systems exhibited significant damage under testing. But the concrete density variation of RPC was larger, especially in those containing steel fibers, as shown in Table 4. It is evident from Table 7 that total concrete porosity was generally greater in systems with steel fibers and lower when made with basalt ones.

The data generated from compressive tests of ordinary and high-strength concrete reported in Table 8 were inside reasonable performance parameters with acceptable scatters. The plots of Poisson’s ratio in Figure 3 did not indicate significant variability from the presence of fibers in the concrete samples. But Young’s modulus plots in Figure 4 were more separated and did illustrate the effect of steel fibers on the RPC performance. 

The connector and the concrete strength are the main factors affecting the behavior of shear connections at the steel-concrete interface in composite member. As actual design methods are based on the test results of studs embedded in normal strength concrete, the load-slip behavior and the shear capacity in higher strength fiber-reinforced concrete were necessary to evaluate the appropriate design. In this study, the tests were conducted by employing six specimens, which differed in material properties of the concrete and stud, as shown in Figure 6, Figure 7 and Figure 8. Experimental push-out tests were used to evaluate both the shear capacity and the load-slip curves of the connection, given in Figure 9 and Figure 10. In the ordinary concrete samples, the cracks were appeared on the surface slightly, concentrated mainly around the studs, because connectors failed by shear, as shown in Figure 9. The samples with connectors cast with RPC in the square holes were damaged by earlier cracking of the normal concrete region, as shown in Figure 10, at a significantly higher ultimate load.

The results of the finite element models were compared with push-out tests and values provided by testing. The graphical overviews in Figure 9 and Figure 10 allowed observing negligible differences of test and theoretically stated outcomes. Besides, the failure modes were appeared pretty similar.

## 5. Conclusions

Two reactive powders concrete reinforced with the steel or basalt fibers and control normal concrete specimens were investigated for structural applications in this study. High performance fiber-reinforced reactive powder concrete revealed its exceptional mechanical and cracking resistances. Thus, the RPC using fine steel or basalt fiber aggregates could meet the mechanical properties requirements for practice in structural applications after being cured in the ordinary curing condition.

A maximum mean compressive strength of 198.3 MPa at 28 days curing age was produced using finely dispersed steel fibers. Compared to the control mix of normal concrete, a 33% larger compressive strength was achieved. Nevertheless, basalt fiber reinforcement of concrete with correspondingly mean values of 135.6 MPa had a less significant impact on the compressive RPC strengths, but with better resistance in an aggressive environment. The flexural strengths of the RPC were, in all cases, greater than those of normal concrete. 

Fiber-reinforced concrete is characterized by a dense microstructure and thus very high strength as well as excellent durability properties. This enables a material saving and weight-reduced, slim construction method that opens up completely new possibilities and areas of application predominantly in precast constructions. But RPC is today still relatively expensive. Besides, a combination with normal concrete may minimize the construction cost. Especially, the shear connection of composite systems can be designed using two types of concrete. The deck slabs could be cast as commonly of normal strength concrete, while the adjacent space of connector location could use higher performance concrete. Executed push-out specimen testing proved to describe suitably shear connection behavior as well as the resistance of shear studs and concrete slab. Besides, post-cracking tensile capacity and pseudo-ductility with work-hardening behavior, accompanied by multiple micros-cracks, were observed in experimental and theoretical studies. This new hybrid connection provided outstanding strength and resistance to transfer shear at the steel-concrete interface. This type of innovative shear connection at the steel-concrete interface could be attractive, especially in the case of precast decks of building or bridge composite structures. The mutual assembly of two different structural parts was simplified and executed faster.

Numerical models making use of the finite element method were developed to simulate the behavior of shear connection in composite structures of steel and concrete of different forms. The comparison with experiments confirmed that these analytical tools were able to describe the real behavior of composite structures, including concrete cracking, crushing, and reinforcement yielding. Therefore, the above procedures may potentially provide more powerful means in practical design.

## Figures and Tables

**Table 1:** Chemical composition of reactive powder concrete (RPC) components (wt.%).

Component	SiO_2_	Fe_2_O_3_	Al_2_O_3_	CaO	MgO	SO_3_	Na_2_O
Cement	21.83	2.00	4.38	65.68	0.93	3.29	0.29
Silica fume	93.3	2.3	1.5	1.0	-	-	-
Quartz powder	99.0	0.05	0.29	<0.1	<0.1	-	0.2
Quartz sand	98.6	0.03	0.75	-	-	-	-

**Table 2:** Composition of RPC mixes (proportion to cement).

Component	C	S	B
Cement	1.00	1.00	1.00
Silica fume	0.20	0.20	0.20
Quartz powder	0.12	0.12	0.12
Quartz sand	1.03	1.03	1.03
Superplasticizer	0.025	0.025	0.025
Steel fibers (kg/m^3^)	-	236	-
Basalt fibers (kg/m^3^)	-	-	3
water cement ratio (w/c)	0.24	0.24	0.24

**Table 3:** Concrete mix flow diameter (mm).

Mix Type	Flow Diameter (mm)
C no fibers	240
S steel fibers	250
B basalt fibers	230

**Table 4:** RPC density (kg/m^3^).

Mix Type	Density (kg/m^3^)
C no fibers	2320
S steel fibers	2590
B basalt fibers	2280

**Table 5:** Results of the compressive and flexural strength tests of RPC (MPa).

RPC Type	Compressive Strength (MPa )		
	2 Days	7 Days	28 Days
C	105.0	114.9	149.6
S	145.4	167.5	198.3
B	88.7	112.2	135.6
	**Flexural Strength (MPa)**		
C	15.8	18.9	21.9
S	48.7	50.8	52.6
B	16.2	20.5	23.2

**Table 6:** RPC concrete frost resistance (kg/m^2^)

RPC Type	Scaling (kg/m^2^)
C	0.0002
S	0.0007
B	0.0005

**Table 7:** RPC concrete total porosity and percentage of different sized pores.

RPC Type	Total Porosity (%)	Percentage of Pores (%)				
		<20 nm	20–200 nm	200–2000 nm	2000–20,000 nm	>20,000 nm
C	4.4	77.1	8.0	3.6	2.5	8.2
S	12.4	15.7	77.0	3.1	0.5	3.4
B	5.4	77.2	8.0	0	0	14.9

**Table 8:** The result of concrete compressive strength measurement.

Specimen Type	Dimensions			Unit Mass	Ultimate Force	Compressive Strength
	Contact Area		Height			
	(mm)	(mm)	(mm)	(kg/m^3^)	(kN)	(N/mm^2^)
1 B-27-06	154.6	150.5	150.3	2390	3014.05	129.54
2 B-25-06	151.5	150.0	150.1	2400	2510.00	110.45
3 B-25-06	153.8	150.1	150.0	2390	3168.93	137.27
4 S-27-06	150.1	149.5	150.0	2158	2945.47	131.26
5 S-27-06	150.1	150.2	150.0	2208	2893.40	129.46
6 S-27-06	150.1	150.2	150.2	2263	2960.67	132.47
7 C-25-06	147.1	148.9	149.2	2382	697.5	31.87
8 C-25-06	149.2	146.8	150.0	2398	682.5	31.16
9 C-25-06	148.8	147.7	150.2	2346	674.9	30.71
